# Health Service Development and Planning for Autism in Egypt

**DOI:** 10.1192/j.eurpsy.2023.1513

**Published:** 2023-07-19

**Authors:** H. Hasan, M. Elhamshary

**Affiliations:** 1 Neuropsychiatry, Helwan University - Faculty of Medicine, Cairo, Egypt; 2 Cumbria, Northumberland, Tyne and Wear NHS foundation trust, Newcastle, United Kingdom

## Abstract

**Introduction:**

Prevalence of Autism Spectrum Disorder (ASD) is 33.6% among children with developmental disabilities in Egypt. Children with ASD have unique needs & interventions must be individualized for successful outcomes. Many systems are involved to provide services or funding resources to assist patients with ASD & their families. Coordination of multiple services is a challenge for families.

**Objectives:**

To understand the changing needs of the expanding autism population.

This effort would be intended for policymakers, service providers, community organizations & advocacy groups to better understand & address the needs of children with autism in Egypt. Needs assessment countrywide would be the first step in developing system of care for these children.

**Methods:**

Surveying of children with autism & their families should be done to assess how or if the needs of children with autism & their families are met along with surveying service providers on the quality of service provided for children with autism.

A questionnaire will be designed for the families to assess the availability of mental health services for ASD patients and its accessibility along with its efficiency & meeting their expectations as regards appropriate care & support, in addition the role of these specialized services in promoting the understanding of the families of ASD patients. Furthermore assessing provision of appropriate primary health care services that are ready to accommodate ASD patients’ special conditions as well as secondary & tertiary services.

Another questionnaire would be provided to mental health professionals dealing with ASD patients, it would include providers at all levels of service like child psychiatrists, general psychiatrists, behavioral therapists, psychologists, nurses, social workers & administrators of services. This questionnaire would assess presence of resources & appropriate management, available funding & if it meets the needs of services, appropriate training for professionals & service providers, coordination between facilities, barriers & limitations they face in their work as well as obtaining their suggestions to enhance the services provided.

**Results:**

All the data will be collected & revised for completeness. Statisticians & community health professionals will be consulted for helping empower the study & guiding our field work. Clustering of data on geographical basis would help understand & prioritize areas of need as well.

**Conclusions:**

To develop system of caring for autism in Egypt we should look at whether Egyptians living with autism are getting the services they need. We should identify barriers to accessing services & examine if the families of children with autism consider the services they do receive to be effective. We believe Egyptians with autism & their families are struggling to find the services they need & are often dissatisfied with the services that are provided.

**Disclosure of Interest:**

None Declared

